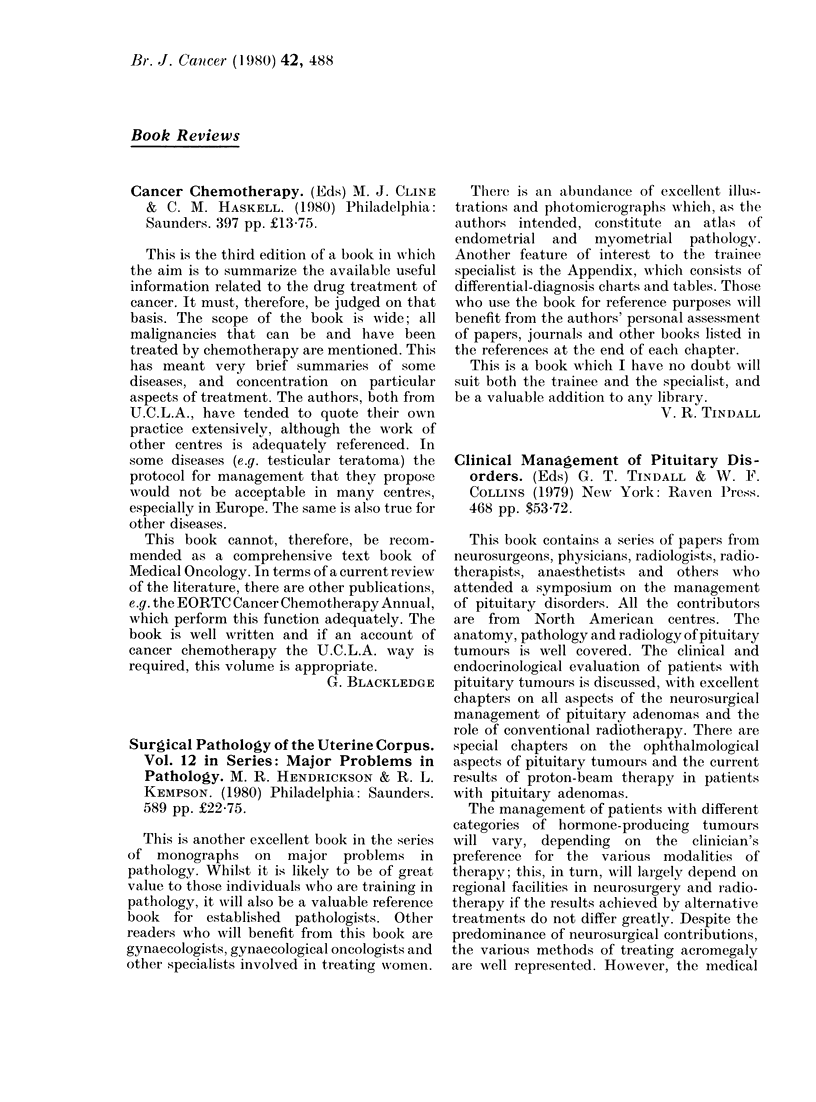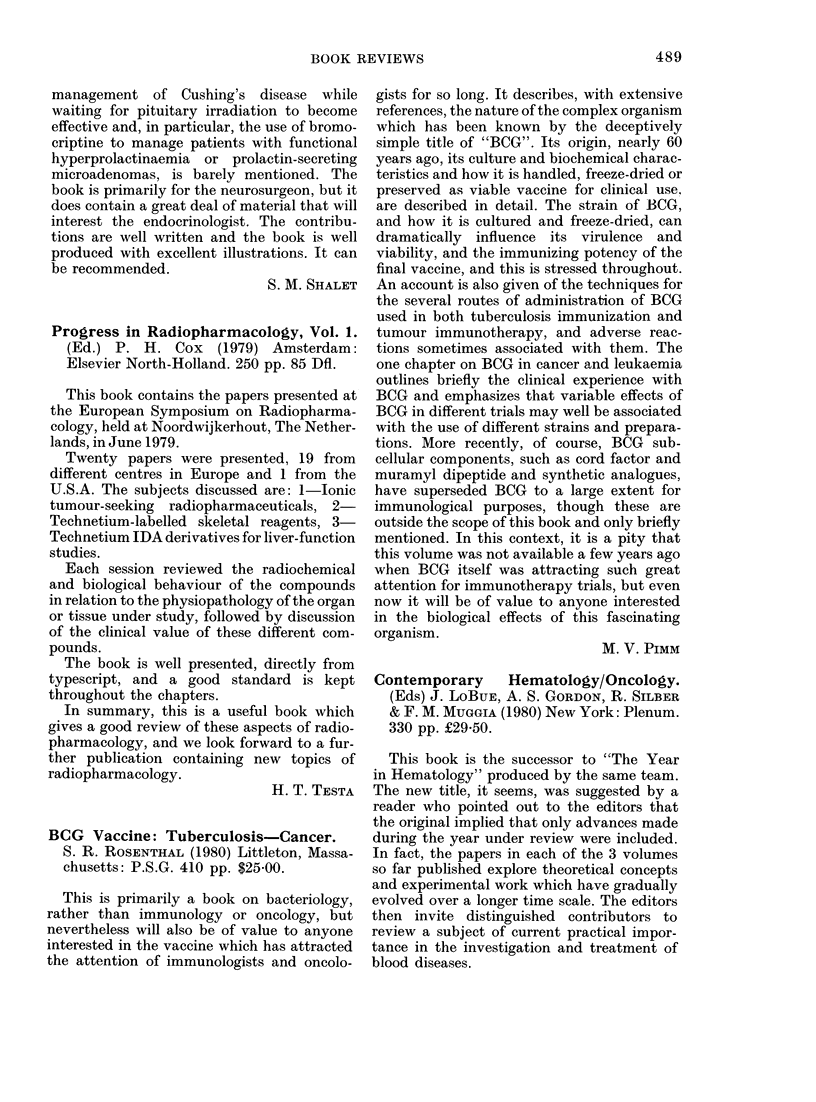# Clinical Management of Pituitary Disorders

**Published:** 1980-09

**Authors:** S. M. Shalet


**Clinical Management of Pituitary Disorders.** (Eds) G. T. Tindall & W. F. Collins (1979) New York: Raven Press. 468 pp. $53·72.

This book contains a series of papers from neurosurgeons, physicians, radiologists, radiotherapists, anaesthetists and others who attended a symposium on the management of pituitary disorders. All the contributors are from North American centres. The anatomy, pathology and radiology of pituitary tumours is well covered. The clinical and endocrinological evaluation of patients with pituitary tumours is discussed, with excellent chapters on all aspects of the neurosurgical management of pituitary adenomas and the role of conventional radiotherapy. There are special chapters on the ophthalmological aspects of pituitary tumours and the current results of proton-beam therapy in patients with pituitary adenomas.

The management of patients with different categories of hormone-producing tumours will vary, depending on the clinician's preference for the various modalities of therapy; this, in turn, will largely depend on regional facilities in neurosurgery and radiotherapy if the results achieved by alternative treatments do not differ greatly. Despite the predominance of neurosurgical contributions, the various methods of treating acromegaly are well represented. However, the medical management of Cushing's disease while waiting for pituitary irradiation to become effective and, in particular, the use of bromocriptine to manage patients with functional hyperprolactinaemia or prolactin-secreting microadenomas, is barely mentioned. The book is primarily for the neurosurgeon, but it does contain a great deal of material that will interest the endocrinologist. The contributions are well written and the book is well produced with excellent illustrations. It can be recommended.

S. M. SHALET